# Case Report: Rethinking pulmonary arterial hypertension: immune and metabolic adaptations in a 34-year case of insidious progression

**DOI:** 10.3389/fcvm.2026.1727803

**Published:** 2026-02-19

**Authors:** Bronwen Erickson-Keith

**Affiliations:** Independent Researcher, Lynnville, TN, United States

**Keywords:** asplenia, chronic progression, endothelial dysfunction, immune dysregulation, metabolic remodeling, pulmonary arterial hypertension

## Abstract

**Background:**

Pulmonary arterial hypertension (PAH) is traditionally characterized as a rapidly progressive, fatal disease. Yet increasing evidence suggests that immune dysregulation, endothelial injury, and metabolic adaptation can sustain subclinical disease for decades before recognition.

**Case presentation:**

We report a 73-year-old U.S. Army veteran with terminal PAH whose symptoms began in 1972—nearly fifty years before diagnosis. Despite a childhood splenectomy and extensive exposure to pulmonary toxins during a 21-year career in military aviation (JP-4/JP-8 fuels, asbestos, burn pits, and talc pleurodesis), no follow-up imaging was performed after retirement in 1992. After a 24-year gap, a 2016 chest x-ray revealed pulmonary artery enlargement consistent with pulmonary hypertension, but the finding was not pursued until 2021, when right-heart catheterization confirmed severe PAH with pulmonary artery pressures of 82/29 mm Hg (mean ≈47 mm Hg). A transthoracic echocardiogram in October 2025 demonstrated an estimated right-ventricular systolic pressure (RVSP) of 66 mm Hg with right-atrial pressure 8 mm Hg, preserved biventricular function, normal ejection fraction (LVEF 68%), mild concentric left-ventricular hypertrophy, and only trace tricuspid and mitral regurgitation. Brain natriuretic peptide (BNP) levels remained normal, suggesting right-ventricular metabolic adaptation rather than decompensation.

**Discussion:**

This case supports a model of *slowly progressive PAH* driven by chronic immune imbalance from asplenia, toxin-induced oxidative stress, and nitric-oxide scavenging by free hemoglobin. Over decades, these mechanisms produced endothelial remodeling while right-ventricular metabolism shifted toward glycolysis (“fetal-like” adaptation), delaying failure. The patient's trajectory parallels post-COVID data linking endothelial inflammation and microvascular injury to delayed pulmonary hypertension.

**Conclusion:**

PAH can evolve silently across decades in individuals with immune or hematologic dysregulation. Recognition of immune-metabolic phenotypes and early screening of asplenic or toxin-exposed populations may enable earlier intervention and alter the disease's natural history.

## 1 Introduction

Pulmonary arterial hypertension (PAH) is a life-limiting disorder characterized by increased pulmonary vascular resistance leading to right-ventricular (RV) failure. Historically, PAH carried a median survival of 2.8 years after diagnosis (1). Advances in pharmacotherapy and diagnostics have extended survival, yet current models still portray the disease as rapidly progressive following symptom onset. Emerging evidence challenges this view, suggesting that subclinical disease may smolder for years or decades before recognition (2, 3, 9).

**Table 1:** Chronology of Key diagnostic findings.

Year	Event/investigation	Findings	Interpretation
1992	Final military imaging	Pleural thickening, blunted costophrenic angles	Post-surgical change; early vascular disease unrecognized
1999	Prostate cancer diagnosis	Prostatectomy (age 47)	Added systemic inflammatory burden
2016	Chest x-ray → CT	Pulmonary artery enlargement noted, dismissed	Early radiographic evidence of PH
2021	Right-heart catheterization	PA 82/29 mm Hg (mean ≈ 47); BNP 75 pg/mL	Severe PAH with preserved RV function
2023	Clinical status	Initiated O₂	Progression but continued compensation
2025	Echocardiogram	RVSP 66 mm Hg (RA 8); LVEF 68%; TAPSE 1.8 cm; trace TR/MR	Persistent severe PH, preserved biventricular function

Recent discoveries in immunology and metabolism provide a new lens for interpreting this heterogeneity. Endothelial injury, chronic inflammation, and mitochondrial reprogramming have been identified as early events in PAH pathogenesis. The COVID-19 pandemic further illuminated parallels between viral-induced endothelial dysfunction and pulmonary vascular remodeling.

Asplenia—a condition of absent splenic function—is a rare but profound modifier of vascular and immune homeostasis. The spleen regulates lymphocyte maturation, filters senescent erythrocytes, and maintains nitric-oxide balance through clearance of free hemoglobin (4, 5). Loss of splenic function results in chronic lymphopenia, heightened oxidative stress, and endothelial injury—mechanisms strongly implicated in pulmonary vascular disease (6). Yet, the contribution of asplenia to long-term PAH evolution remains underexplored.

We present the case of a veteran with terminal PAH whose disease likely originated during military service in the early 1970s and progressed insidiously for decades. This case illustrates how chronic immune dysregulation, environmental exposure, and metabolic adaptation can yield an extended preclinical phase and atypically long survival.

## 2 Case presentation

The patient is a 73-year-old U.S. Army veteran (CW3, Ret.) with a history of trauma-related splenectomy at age 16 and 21 years of active-duty aviation service (1971–1992), during which he was exposed to JP-4/JP-8 jet fuels, methyl-ethyl-ketone (MEK), asbestos, burn pits, and underwent talc pleurodesis in 1991 following recurrent spontaneous pneumothoraces. During military service, he developed recurrent chest pain, chronic headaches, and exertional intolerance; however, no pulmonary hypertension–specific evaluation was pursued.

Following retirement in 1992, no routine cardiopulmonary imaging or echocardiography was performed for 24 years. In 2016, a chest x-ray demonstrated prominent pulmonary arteries and right-heart border enlargement, interpreted as suggestive of pulmonary hypertension. Subsequent CT imaging did not confirm this finding, and no additional cardiopulmonary evaluation was undertaken.

Definitive diagnosis was established in 2021 via right-heart catheterization, which revealed severe pulmonary arterial hypertension with pulmonary artery pressures of 82/29 mm Hg (mean ≈47 mm Hg) (11). Despite these markedly elevated pressures, right-ventricular enlargement was mild, systolic function was preserved, and brain natriuretic peptide (BNP) levels remained within normal limits (75 pg/mL). Supplemental oxygen therapy was initiated in 2023.

A transthoracic echocardiogram performed in October 2025 demonstrated an estimated right-ventricular systolic pressure (RVSP) of 66 mm Hg (right atrial pressure 8 mm Hg), preserved biventricular function with a left-ventricular ejection fraction (LVEF) of 68%, TAPSE of 1.8 cm, mild concentric left-ventricular hypertrophy, trace tricuspid and mitral regurgitation, and no pericardial effusion—findings consistent with sustained hemodynamic burden and preserved right-ventricular compensation.

Laboratory trends over time revealed chronically low lymphocyte counts, low-normal red blood cell indices, and intermittent hyponatremia and hypochloremia, supporting the presence of chronic inflammation and neurohormonal dysregulation.

### 2.1 Diagnostic assessment

Diagnostic evaluation was substantially delayed due to the absence of routine cardiopulmonary surveillance following military retirement and the non-specific nature of early symptoms, including exertional intolerance, chronic headaches, and intermittent chest discomfort. Although pulmonary artery enlargement was visible on chest x-ray in 2016, subsequent CT imaging failed to confirm pulmonary hypertension, and echocardiographic assessment was not pursued at that time. The diagnosis of severe pulmonary arterial hypertension was ultimately confirmed in 2021 by right-heart catheterization (10). This diagnostic trajectory highlights the insidious progression of disease and historical under-recognition of slowly progressive PAH phenotypes.

### 2.2 Therapeutic intervention

At the time of diagnosis, therapeutic options were limited due to the advanced stage of disease and prolonged preclinical progression prior to recognition. Management focused primarily on supportive care, including initiation of supplemental oxygen therapy in 2023 and longitudinal cardiopulmonary monitoring. Pharmacologic therapy with a phosphodiesterase-5 inhibitor (tadalafil 5 mg twice daily) was initiated as part of standard pulmonary arterial hypertension management; however, the late timing of intervention and underlying comorbidities constrained the potential for disease modification. Treatment decisions emphasized symptom management, preservation of functional capacity, and ongoing assessment rather than reversal of established pulmonary vascular remodeling.

### 2.3 Follow-up and outcomes

Following diagnosis, the patient underwent regular clinical follow-up and echocardiographic surveillance. Despite persistently elevated pulmonary pressures, right-ventricular function remained preserved, with stable ejection fraction and consistently normal BNP levels on serial evaluation. Oxygen therapy improved exertional tolerance but did not fully alleviate symptoms. The extended period of compensation despite severe hemodynamic burden underscores adaptive right-ventricular remodeling and metabolic flexibility (17, 18).

### 2.4 Patient perspective

The patient described prolonged frustration related to decades of unexplained symptoms prior to diagnosis. He noted that the absence of clear explanations over many years contributed to uncertainty and delayed care. The patient expressed hope that publication of this case might assist clinicians in recognizing similar patterns earlier, particularly in individuals with long-standing, exposure-related cardiopulmonary symptoms, and reduce diagnostic delays for others with slowly progressive pulmonary vascular disease.

#### 2.4.1 Patient consent

Written informed consent was obtained for publication of this case and all accompanying data and figures.

## 3 Discussion

### 3.1 Pathophysiologic framework

**Figure 1:**
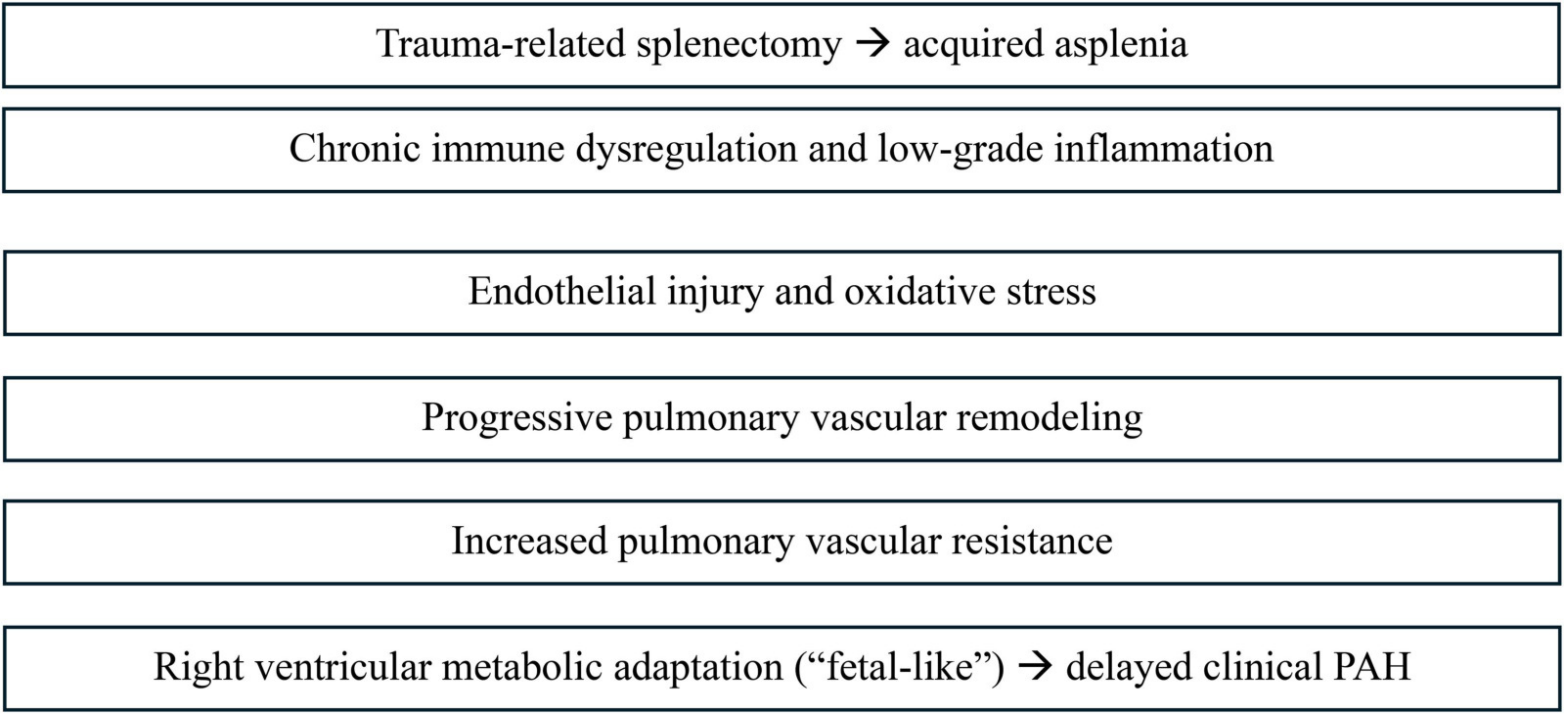
Proposed immune–metabolic mechanism underlying slowly progressive pulmonary arterial hypertension in the setting of trauma-related splenectomy and chronic environmental exposure. Acquired asplenia may contribute to persistent immune dysregulation and low-grade inflammation which, in combination with endothelial injury and oxidative stress from long-term exposures, promotes gradual pulmonary vascular remodeling. Adaptive right ventricular metabolic shifts (“fetal-like”) may preserve function despite increasing hemodynamic burden, allowing prolonged subclinical disease prior to overt clinical manifestation.

This case exemplifies a *slowly progressive, immune-mediated phenotype* of pulmonary arterial hypertension (PAH). The patient's decades-long latency between symptom onset and hemodynamic confirmation reflects a pathobiologic process that began with immune dysregulation from asplenia, was amplified by environmental toxin exposure, and was sustained by endothelial dysfunction and metabolic reprogramming of the right ventricle (RV). A schematic overview of the proposed immune–metabolic mechanisms underlying slowly progressive PAH is provided in Figure 1.

### 3.2 Proposed immune–metabolic mechanism of slowly progressive pulmonary arterial hypertension

#### 3.2.1 Asplenia-driven immune dysregulation

The spleen is a critical regulator of immune and hematologic homeostasis. Its absence produces chronic lymphopenia, altered B- and T-cell maturation, and impaired clearance of senescent erythrocytes (4). Accumulation of damaged red blood cells increases circulating free hemoglobin, which avidly scavenges nitric oxide (NO), reducing pulmonary vasodilation and predisposing to microthrombi and smooth-muscle proliferation (5). Persistent low-grade inflammation, driven by elevated cytokines such as IL-6 and TNF-α, promotes vascular remodeling—a hallmark of PAH (7).

In this patient, serial complete blood counts over five decades documented lymphopenia and low-normal hemoglobin, confirming chronic immune imbalance consistent with an asplenic profile. These hematologic findings likely pre-dated the vascular remodeling observed on imaging.

#### 3.2.2 Environmental and surgical exposures

Service-related exposures to JP-4/JP-8 fuels, MEK, asbestos, and burn-pit particulates (13) added a second tier of endothelial injury through oxidative stress and direct cytotoxicity (8, 19). The 1991 talc pleurodesis, performed to treat recurrent pneumothorax, may have further intensified pleural and vascular inflammation (14). Together, these insults created a milieu of chronic oxidative stress in an individual already primed for inflammatory dysregulation.

#### 3.2.3 Endothelial dysfunction and vascular remodeling

The intersection of hemolysis, inflammation, and toxin-mediated ROS generation produces NO depletion, endothelin-1 up-regulation, and prostacyclin deficiency, shifting the pulmonary vasculature toward vasoconstriction and thrombosis (2). Over time, these changes culminate in medial hypertrophy and intimal fibrosis, increasing pulmonary vascular resistance while the disease remains clinically silent. The patient's 2016 chest x-ray, showing pulmonary artery enlargement, marked the first visible manifestation of these microscopic changes that had likely been evolving for decades.

#### 3.2.4 Right-ventricular metabolic adaptation

Despite a mean pulmonary artery pressure of approximately 47 mm Hg in 2021, the RV remained functionally preserved, suggesting adaptive metabolic remodeling. Contemporary studies demonstrate that the pressure-overloaded RV transitions from fatty-acid oxidation to glucose-based glycolysis, a “fetal-like” energy phenotype that conserves oxygen and delays failure (3). This shift, although less energy-efficient, improves ATP generation under hypoxic stress. The patient's stable BNP values (75 → 64 pg/mL) and normal TAPSE (1.8 cm) support this adaptive, rather than decompensated, state.

#### 3.2.5 Parallel insights from post-COVID endothelial injury

The global recognition of post-COVID pulmonary vascular disease has renewed interest in immune-endothelial cross-talk. SARS-CoV-2 induces endotheliosis, microthrombi, and long-term remodeling through cytokine activation and mitochondrial stress—mechanisms mirroring those observed here. Asplenic patients may represent an analogous model of chronic immune activation leading to sustained endothelial damage. Both scenarios underscore that PAH can arise from persistent, systemic vascular inflammation rather than a single acute event.

#### 3.2.6 Clinical and research implications

This case challenges the long-standing assumption that PAH is invariably acute in onset and rapidly fatal. Instead, it suggests a continuum of disease, beginning with subclinical immune and metabolic alterations that may persist for decades before hemodynamic thresholds are reached. Clinically, such patients may present with non-specific symptoms—progressive exertional dyspnea, dizziness or syncope, and mild exercise intolerance—long before overt RV strain (15, 16, 20) (Table 1).

Early recognition requires:

- Vigilance for pulmonary vascular changes in asplenic or chronically inflamed individuals.
- Periodic echocardiography following toxin or hypoxia exposure.
- Integration of immune biomarkers (IL-6, TNF-α) and metabolic imaging (FDG-PET) into longitudinal monitoring.

From a research perspective, prospective studies exploring the interplay between immune dysregulation, endothelial injury, and RV metabolism could clarify why some patients exhibit protracted compensation. Investigating asplenia and chronic hemolysis as independent risk factors for PAH may also identify a preventable subset of disease.

## 4 Limitations

This report represents a single, retrospective case and cannot establish causality. Historical data were reconstructed from military, VA, and civilian records spanning five decades, and some laboratory or imaging details were unavailable. Hemodynamic measurements prior to 2021 were not obtained, limiting temporal precision in assessing disease onset and progression. In addition, molecular or genetic testing—such as BMPR2 or other heritable PAH markers—was not performed. Nevertheless, the comprehensive longitudinal record and integration of physiologic, immunologic, and metabolic evidence offer a rare window into a slowly progressive phenotype of pulmonary arterial hypertension (PAH). The interpretation presented here is consistent with current mechanistic frameworks and supported by relevant literature, but prospective validation is needed.

## 5 Conclusion

This case challenges the conventional paradigm of pulmonary arterial hypertension as an acutely progressive disease and supports a model of slow, immune-mediated vascular remodeling culminating in advanced PAH after decades of compensation. The patient's history of asplenia, chronic inflammation, environmental toxin exposure, and delayed diagnosis align with a systems-level pathophysiology in which immune dysregulation drives endothelial dysfunction and gradual metabolic adaptation within the right ventricle.

His preserved ventricular function despite pressures exceeding 80 mm Hg (12) underscores the capacity of the human heart to remodel metabolically—shifting toward glycolytic energy utilization to preserve performance under sustained stress. Such adaptive “fetal-like” physiology may explain prolonged survival in select patients and highlights the importance of early recognition before decompensation occurs.

Future research should focus on:

1. Defining asplenia and chronic hemolysis as independent risk factors for PAH.
2. Elucidating immune-metabolic biomarkers predictive of slow-progressing disease.
3. Developing screening strategies for veterans and other toxin-exposed populations.

Understanding PAH as a spectrum disorder—with both rapidly progressive and indolent forms—may transform prevention and management by shifting emphasis from late-stage intervention to early, immune-informed surveillance.

## Data Availability

The datasets presented in this article are not readily available because the dataset contains identifiable patient health information and cannot be shared publicly due to privacy and confidentiality regulations. Summarized data relevant to the findings are included within the article and tables. Additional details are available from the author upon reasonable request and with appropriate ethical approval. Requests to access these datasets should be directed to the corresponding author.
